# Neoadjuvant trials in early breast cancer: pathological response at surgery and correlation to longer term outcomes – what does it all mean?

**DOI:** 10.1186/s12916-015-0472-7

**Published:** 2015-09-22

**Authors:** Helena Earl, Elena Provenzano, Jean Abraham, Janet Dunn, Anne-Laure Vallier, Ioannis Gounaris, Louise Hiller

**Affiliations:** Department of Oncology, University of Cambridge, Cambridge, UK; NIHR Cambridge Biomedical Research Centre and Cambridge Experimental Cancer Medicine Centre, Cambridge, UK; Cambridge Breast Research Unit, Cambridge, UK; Cambridge University Hospital NHS Foundation Trust, Cambridge, UK; Warwick Clinical Trials Unit, University of Warwick, Coventry, UK; Cancer Research UK Cambridge Institute, Cambridge, UK

**Keywords:** Early breast cancer, Longer-term outcomes, Neoadjuvant trials, Pathological response

## Abstract

**Background:**

Neoadjuvant breast cancer trials are important for speeding up the introduction of new treatments for patients with early breast cancer and for the highly productive translational research which they facilitate. Meta-analysis of trial data shows clear correlation between pathological response at surgery after neoadjuvant chemotherapy and longer-term outcomes at an individual patient level. However, this does not appear to be present on individual trial level analysis, when correlating improved outcome for the investigational arm for the primary endpoint (pathological response) with longer-term outcomes.

**Discussion:**

The correlation between pathological response and longer-term outcomes in trials is dependent on many factors. These include definitions of pathological response, both complete and partial; assessment methods for pathological response at surgery; subtype and prognosis of breast cancer at diagnosis; number of patients recruited; adjuvant treatments; the mechanism of action of the investigational drug; the length of follow-up at the time of reporting; the definitions used in longer-term outcomes analysis; clonal heterogeneity; and new adaptive trial designs with additional neo/adjuvant treatments. Future developments of neoadjuvant breast cancer trials are discussed. With so many factors influencing the correlation of longer-term outcomes for trial-level data, we conclude that the main focus of neoadjuvant trials should remain the primary endpoint of pathological response.

**Summary:**

Neoadjuvant breast cancer trials are very important investigational studies that will continue to increase our understanding of the disease and offer the potential of more rapid introduction of new treatments for women with high-risk early breast cancer. In the future, we are likely to see both novel trial designs adopted in the neoadjuvant context and modifications of neo/adjuvant treatments for pathological non-responders within clinical trials. Both of these have the intention of improving longer-term outcomes for patients who do not have a good pathological response to first-line neoadjuvant treatment. If successful, these developments are likely to reduce further any positive correlation between pathological response and longer-term outcomes.

## Background

**Fig. 1:**
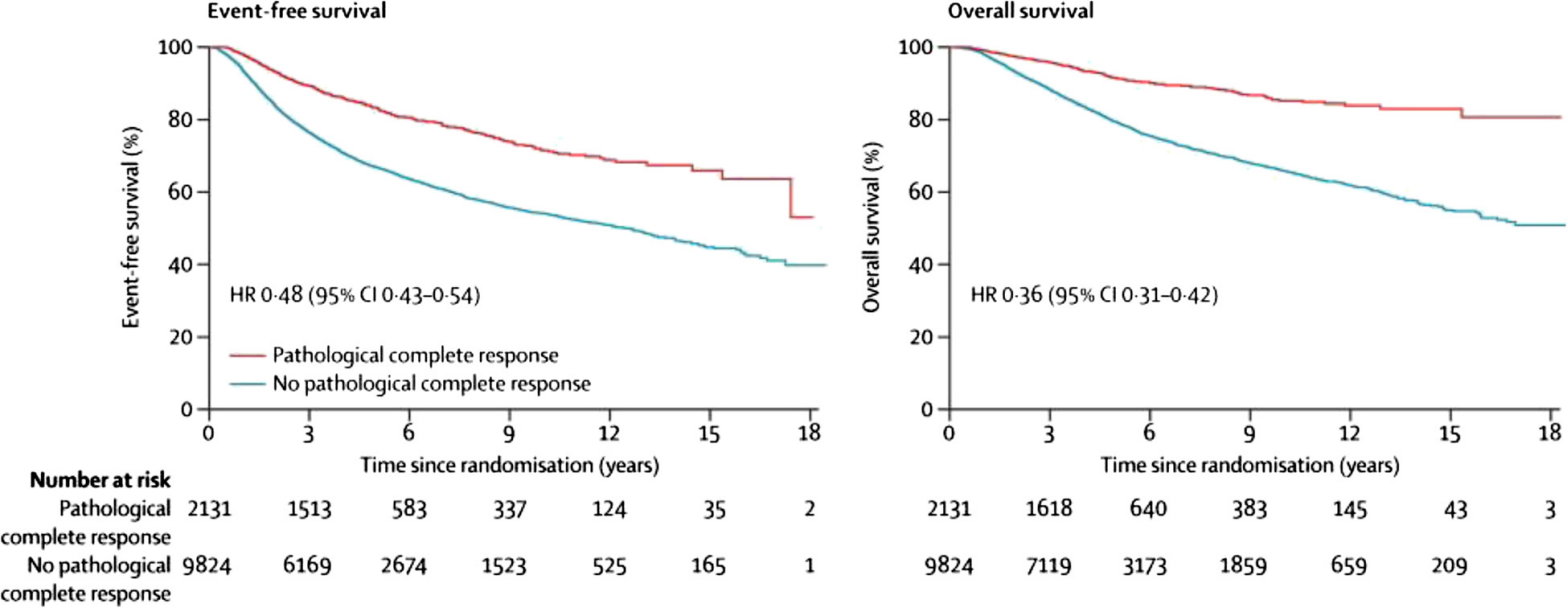
Associations between pathological complete response and event-free survival and overall survival. The ypT0/is ypN0 definition of pathological complete response was used (i.e. absence of invasive cancer in the breast and axillary nodes, irrespective of ductal carcinoma in situ). *CI* confidence interval, *HR* hazard ratio. (Reproduced with permission from *The Lancet*, Cortazar et al. [5]: License Number 3666940645625)

Neoadjuvant treatment of early breast cancer has many advantages both for patients and for the ‘rich’ track of clinical, translational and scientific research that can be carried out. Published evidence confirms a reduction in mastectomy rates with increasing use of neoadjuvant therapy both on a population [1] and individual trial basis [2–4]. The relationship between pathological response and longer-term outcome in women with early breast cancer receiving neoadjuvant systemic therapy is highly complex and its' dependencies are multifactorial. This opinion article discusses the relationship between the two. A recent meta-analysis of neoadjuvant breast cancer trials [5] and a meta-regression of trials data [6] have confirmed in just short of fifteen thousand women the robust relationship between achieving a pathological response (particularly a complete pathological response, pCR) and improved longer-term outcomes on an individual patient level (Fig. 1). We will explore the factors that, for the trial level question, influence the relationship between the primary endpoint (pCR) and the longer-term outcomes. We also explore why, on an individual trial level analysis, any correlation between improved outcome for the investigational trial arm—in terms of the primary endpoint (pathological response)—and longer term outcomes seems more challenging to establish [5–8], with very few exceptions [9, 10]. Nevertheless, our opinion is that neoadjuvant treatments and trials should retain their place at the forefront of research and treatment for high-risk early breast cancer.

## Discussion

### What do we mean when we say pathological complete response?

Neoadjuvant clinical trials, using pCR as an evaluable endpoint, have gained acceptance as a means of the initial evaluation of the efficacy of new agents. However, comparison of outcomes of these trials is currently difficult, because different trials use different definitions of pCR. Many early neoadjuvant trials looked at response in the breast only, and defined pCR as no residual invasive disease in the breast, irrespective of the presence of ductal carcinoma in situ (DCIS) or nodal involvement (ypT0/is ypNx). Some later trials used a more stringent definition that included response in both the breast and the axillary lymph nodes, either allowing the presence of residual DCIS (ypT0/is ypN0) or requiring absence of both invasive disease and DCIS in the breast (ypT0 ypN0).

**Fig. 2:**
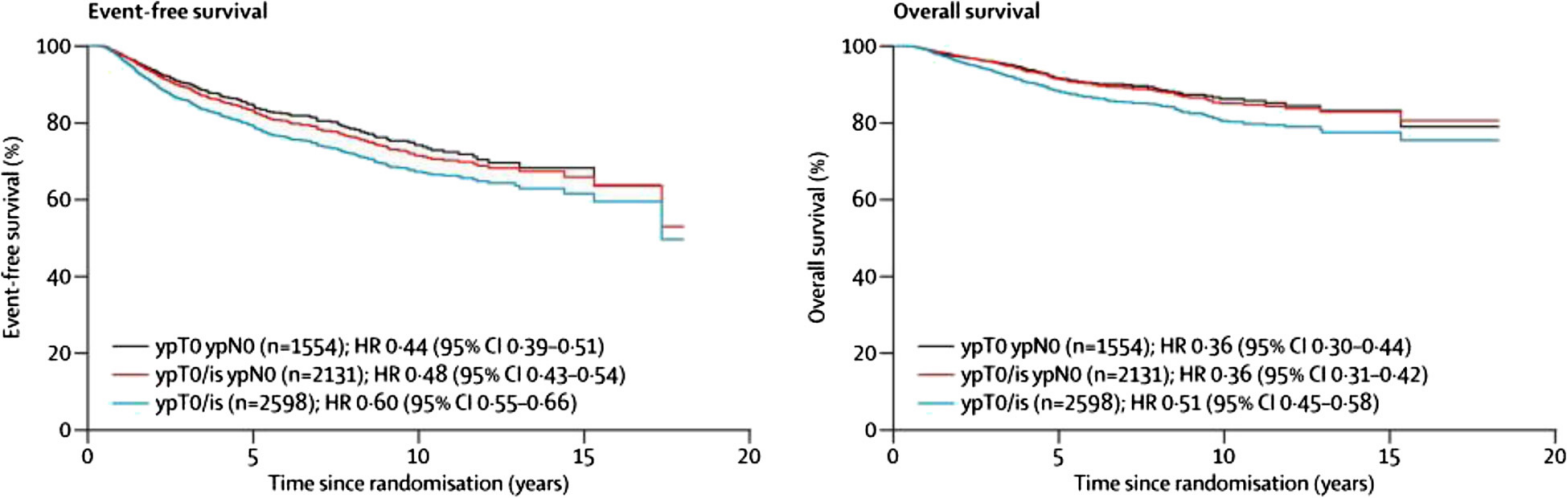
Associations between three definitions of pathological complete response and event-free survival and overall survival. We compared event-free survival and overall survival between patients who did and did not achieve a pathological complete response according to one of three definitions. Patients who did not achieve pathological complete response are not shown. The number of patients who achieved a pathological complete response is listed for each pathological complete response definition. Patients could achieve pathological complete response according to more than one definition. ypT0ypN0 = absence of invasive cancer and in situ cancer in breast and axillary nodes. ypT0/is ypN0 = absence of invasive cancer in breast and axillary nodes, irrespective of ductal carcinoma in-situ. ypT0/is = absence of invasive cancer in breast, irrespective of ductal carcinoma in-situ or nodal involvement. *CI* confidence interval, *HER2* human epidermal growth factor receptor 2, *HR* hazard ratio. (Reproduced with permission from *The Lancet*, Cortazar et al. [5]: License Number 3666940645625)

A meta-analysis of 12 major neoadjuvant randomised trials involving 11,955 patients was undertaken by the Collaborative Trials in Neoadjuvant Breast Cancer (CTNeoBC) Group [5]. They examined the different definitions of pCR, with overall pCR rates of 22 % for ypT0/is ypNx, 18 % for ypT0/is ypN0 and 13 % for ypT0 ypN0. Event-free and overall survival (OS) was found to be significantly worse for the group with residual nodal involvement and similar in the remaining two groups without residual invasive cancer in breast and lymph nodes regardless of the presence or absence of DCIS (Fig. 2). As a result, the US Food and Drug Administration (FDA) have advocated use of either ypT0 ypN0 or ypT0/is ypN0 as definitions of pCR in their guidance on the use of pCR as an endpoint for accelerated approval for agents in the neoadjuvant treatment of aggressive early breast cancer [11, 12].

There is general consensus that the definition of pCR should include axillary lymph node status, because several studies have shown that residual disease in the axillary lymph nodes indicates a worse prognosis, even in the presence of pCR in the breast [13–15]. In both the neo-tAnGo and MD Anderson series, this group represented around 4 % of patients [15, 16]. Potential explanations include sampling error in the breast, due to extensive size or inaccurate localisation of the primary tumour bed, or presence of a resistant sub-clone in the metastasis.

The issue of whether DCIS should be included in the definition of pCR is more contentious. A single-institution cohort study of patients from the MD Anderson Cancer Center showed no difference in survival between patients with and without residual DCIS, similar to the CTNeoBC meta-analysis [5, 17]. However, a pooled analysis of seven prospective neoadjuvant clinical trials performed by the German and Austrian Breast Groups found significantly longer disease-free survival in patients without residual DCIS (hazard ratio [HR] 1.74; *p* < 0.001), with a non-significant trend towards increased OS (HR 1.41, *p* = 0.166) [18].

The definition of pCR used should be clearly stated in the pathology report. Regardless of which definition is used, the presence of residual DCIS and nodal metastasis should be recorded and quantified as per the adjuvant setting.

### Why is histologic assessment and quantification of residual disease important?

**Fig. 3:**
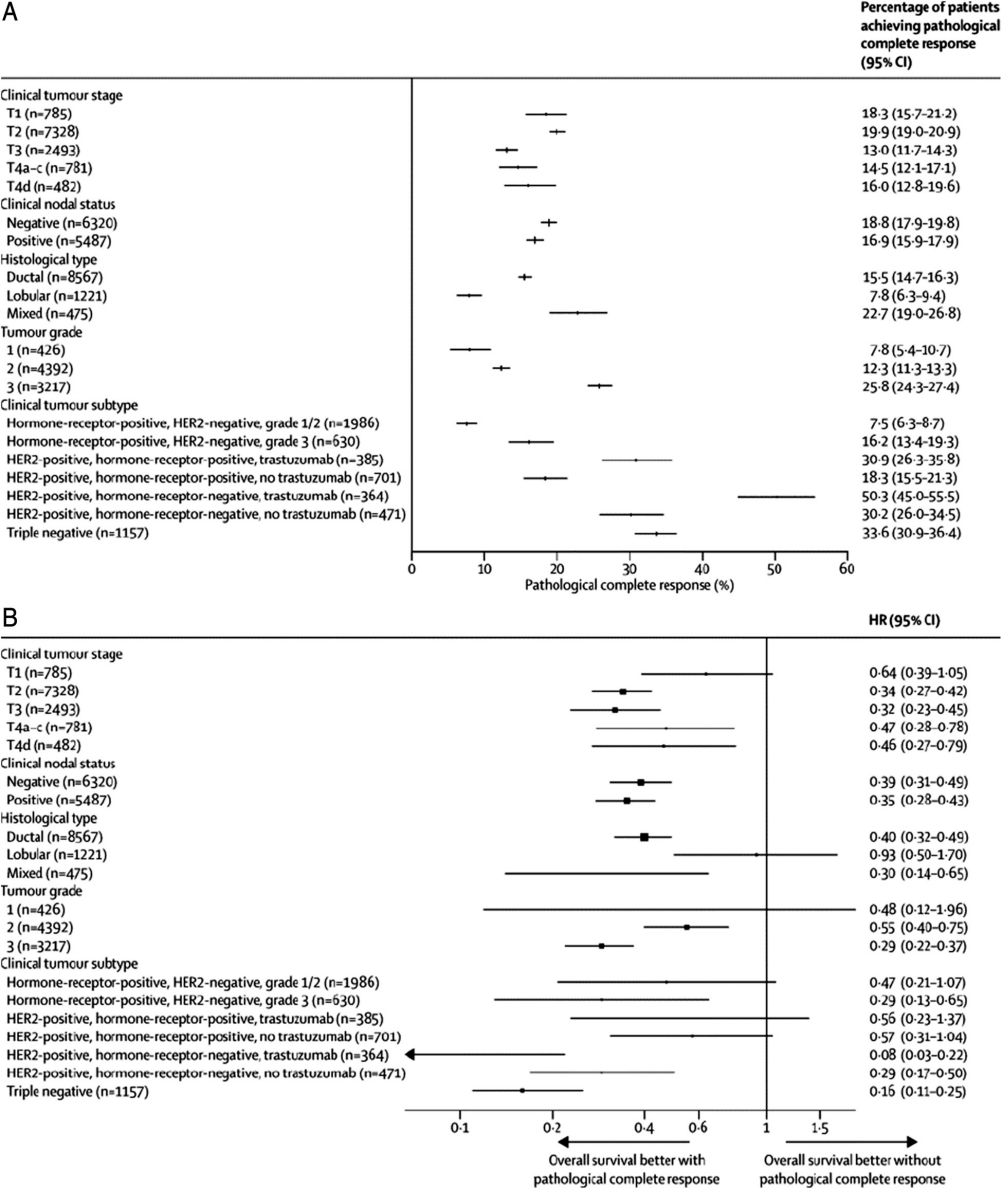
Percentage of patients achieving pathological complete response (**a**) and HRs for overall survival (**b**), by subgroup.Information about clinical tumour stage available for 11 869 patients, about clinical nodal status for 11 807 patients, about histological type for 10,263 patients, about tumour grade for 8035 patients, and about clinical subtype for 5694 patients. ypT0/isypN0 definition of pathological complete response used. No multiplicity adjustment was made. *HR* hazard ratio, *CI* confidence interval, *HER2* human epidermal growth factor receptor 2. (Reproduced with permission from *The Lancet*, Cortazar et al. [5]: License Number 3666940645625)

The likelihood of pCR in breast cancer is heavily influenced by the biological subtype. Breast cancers that are low grade and oestrogen receptor (ER)- and progesterone receptor (PR)-positive and human epidermal growth factor receptor 2 (HER2)-negative have the lowest rates of pCR (<10 %), with a weaker association between not achieving a pCR and survival outcomes. In contrast, HER2-positive/ER-negative and triple-negative (ER/PR/HER2-negative) breast cancers show much higher rates of pCR, with a stronger association between not achieving a pCR and poorer outcome (Fig. 3).

Residual disease includes a broad spectrum of responses, from excellent response with minimal disease measuring only a few millimetres, to no response or even progression of disease on chemotherapy. In order to refine and maximise the prognostic utility of response to neoadjuvant chemotherapy, accurate evidence-based stratification of the group of patients with partial response is essential. To achieve this, there needs to be better standardisation of specimen handling and histological reporting of neoadjuvant breast cancer specimens. The BIG-NABCG Residual Disease Working Group has recently formulated recommendations for the standardised pathological evaluation and reporting of neoadjuvant breast cancer specimens, which will hopefully facilitate accurate comparison of treatment outcomes within and across clinical trials [19]:

1. **Specimen handling:** Specimen handling incorporates macroscopic description, slicing and sampling of the surgical specimen (ideally including samples for translational research). Following neoadjuvant chemotherapy, the residual tumour may be difficult to detect on gross examination, particularly when there has been an excellent clinical response and there may be no macroscopic lesion at all or only a vague area of fibrosis. Good multi-disciplinary team communication, provision of accurate clinical notes, and macroscopic correlation with clinical and imaging findings is essential to ensure the correct area is sampled. Placement of marker clips in the tumour bed before commencement of therapy, even in patients undergoing mastectomy, is invaluable for accurate localisation of the tumour bed.
2. **Grading response:** There are numerous systems in the literature for grading of response post neoadjuvant chemotherapy; these have been reviewed in detail elsewhere [20–23]. The main systems are summarised in Table 1. There are two main approaches to assessment of residual disease post chemotherapy. Sometimes tumour size does not decrease but there may be a marked reduction in tumour cellularity. The first approach involves comparison of the tumour cellularity pre and post treatment, giving an estimate of tumour response to treatment. Systems that use this approach include the Sataloff, Chevallier, Miller-Payne and Pinder systems [21, 24–26]. The presence of response in both the breast and lymph nodes should be noted. Disadvantages are that comparison with the pre-treatment core biopsy is required and this may not always be available, and there is no indication of the extent of residual disease.

**Table 1:** Classification systems of pathological response to neoadjuvant breast cancer treatment

Classification system	Comment
American Joint Committee on Cancer/Union for International Cancer Control staging system 7th edition	
ypT ypN, same categories as for adjuvant setting	No evaluation of response;
	No published data relating current edition definitions to survival outcomes
Chevallier	
Class 1. No invasive carcinoma or DCIS, negative lymph nodes	Class 1 and 2 = pCR (DCIS allowed)
Class 2. DCIS in the breast, no invasive carcinoma, negative lymph nodes	
Class 3. Invasive carcinoma with stromal alteration	
Class 4. Few modifications of tumour appearance	
Sataloff	
Tumour:	T-A includes pCR and minimal residual disease
T-A. Total or near total therapeutic effect	T-A versus other categories associated with survival outcomes
T-B. >50 % therapeutic effect, but less than T-A	
T-C. <50 % therapeutic effect	
T-D. No therapeutic effect	
Nodes:	
N-A. Evidence of therapeutic effect, no metastasis	
N-B. No nodal metastasis or therapeutic effect	
N-C. Evidence of therapeutic effect, but metastasis present	
N-D. Metastatic disease, no therapeutic effect	
Miller Payne	
Grade 1. No reduction in overall cellularity	DCIS allowed for pCR
Grade 2. Minor loss of tumour cells (up to 30 %)	Does not include response in the lymph nodes
Grade 3. 30–90 % reduction in tumour cellularity	Association with survival outcomes
Grade 4. >90 % loss of tumour cellularity	
Grade 5. No malignant cells identifiable; DCIS may be present	
Pinder	
Breast:	DCIS allowed for pCR
1. pCR: (1) no residual carcinoma or (2) no residual invasive tumour but DCIS present.	
2. Partial response: (1) minimal residual disease ( <10 % of tumour remaining) , (2) evidence of response with 10–50 % of tumour remaining or (3) >50 % of tumour cellularity remaining with some features of response present.	
3. No evidence of response to therapy.	
Lymph nodes:	Practical approach which is easy to apply
1. No evidence of metastasis or response.	No published data regarding association with survival outcomes
2. Metastases not present but evidence of response.	
3. Metastasis present with evidence of response.	
4. Metastasis present with no evidence of response.	
Residual Cancer Burden score	
Combines tumour size in two dimensions, average residual cellularity, number of involved nodes and size of largest metastases. Online calculator to generate a continuous numerical index that is subdivided into four classes (0 = pCR, I, II and III).	Quantifies residual disease rather than evaluates response
	Reproducible and relatively easy to apply
	Validated in several independent cohorts
	Significant association with survival outcomes over long term follow up

*DCIS* ducal carcinoma in situ, *pCR* complete pathological response

The Residual Cancer Burden score (RCB) is an alternative approach that quantifies the volume of residual disease remaining following neoadjuvant therapy, rather than actual response per se [27]. The score is derived from the size of the residual invasive cancer measured in two dimensions, residual tumour cellularity and proportion of in situ disease estimated by review of up to five slides representing the maximum tumour dimension, the number of involved nodes and the size of largest metastasis. The values can be entered into an online calculator available at [28] that provides the RCB score and class (0–III). The RCB is relatively simple to apply, reproducible, and has been validated with longer-term clinical follow-up data [29]. The RCB score has been advocated as the preferred system for use in clinical trials [19], because partial response is calculated as a continuous variable that may provide more information regarding the relationship between pathological residual disease and clinical outcomes than looking at pCR alone.

To illustrate how the different definitions of pathological response can alter the headline results for a trial, we include here results from our recently published ARTemis trial [4], demonstrating results for ypT0/is ypN0, ypT0/is and ypT0/is plus minimal residual disease in the breast only (Table 2) for different groups of patients. The most important difference may be for those patients with pCR in the breast but with disease in axillary lymph nodes, because these patients seem to have longer-term outcomes more in keeping with those of patients who do not achieve a pCR in the breast. This means that if they are included in the pCR longer-term outcome correlation an additional bias will be introduced.

**Table 2:** Pathological complete response and minimal residual disease in response to D-FEC and Bev + D-FEC – ARTemis trial

	D → FEC	Bev + D → FEC	
	% (95 % CI)	% (95 % CI)	*p* value^a^
pCR in all breast tumours and absence of disease in all removed axillary lymph nodes (ypT0/Tis ypN0)^b^	(n = 66/393)	(n = 87/388)	0.03
	17 % (13–21 %)	22 % (18–27 %)	
ER negative (Allred 0–2) (n = 241)	31 % (23–40)	45 % (36–55)	
ER weakly positive (Allred 3–5) (n = 74)	30 % (16–47)	51 % (34–68)	
ER strongly positive (Allred 6–8) (n = 466)	7 % (4–11)	6 % (3–10)	
pCR in all breast tumours (ypT0/Tis)	(n = 76/394)	(n = 99/388)	0.02
	19 % (16–24)	26 % (21–30)	
ER negative (Allred 0–2) (n = 241)	34 % (25–43)	49 % (39–58)	
ER weakly positive (Allred 3–5) (n = 75)	39 % (24–57)	59 % (42–75)	
ER strongly positive (Allred 6–8) (n = 466)	9 % (5–13)	8 % (5–12)	
pCR or minimal residual disease in all breast tumours	(n = 114/394)	(n = 138/388)	0.03
	29 % (25–34 %)	36 % (31–41 %)	
ER negative (Allred 0–2) (n = 241)	44 % (35–54)	58 % (49–67)	
ER weakly positive (Allred 3–5) (n = 75)	50 % (33–67)	70 % (53–84)	
ER strongly positive (Allred 6–8) (n = 466)	18 % (13–23)	19 % (14–24)	

^a^Adjusted for the five stratification variables (age [≤50, >50 years old], ER status [negative, weakly positive, strongly positive], tumour size [≤50, >50 mm], clinical involvement of axillary nodes [no, yes], and inflammatory or locally advanced disease [no, yes])

^b^Primary endpoint for the ARTemis trial

In summary, in the meta-analysis of neoadjuvant chemotherapy trials carried out by the US FDA, the definition of pCR that is endorsed is absence of residual invasive disease in the breast and axillary lymph nodes either ypT0 ypN0 or ypT0/is ypN0. This is the accepted definition that should be used in reporting neoadjuvant clinical trials in the future. The definition of the degree of residual disease in the breast and axillary lymph nodes is not uniformly agreed, although the RCB [27] score described here is being increasingly used.

### What influences the relationship between pCR and longer-term outcomes in neoadjuvant breast cancer?

#### Definition of pCR and review

How the definition of pathological response used within neoadjuvant trials is arrived at is rarely included in the reports. An ideal would be to have a central pathological review of each case in full although this is unlikely to be practical in the timeframes and with the samples available. In Neo-tAnGo [7] and ARTemis [4], we carried out a two-reader blinded report review as described in Provenzano et al. [23], with further review of the reports when the two readers disagreed to obtain consensus. Our neoadjuvant group feels confident that this is a robust enough strategy for determining pCR for each patient. We will have the opportunity to test this in the ARTemis trial, in which we have a completed report review [4] and a central pathology review in progress.

It is essential to include the definition of pCR that was used within a trial in the paper, and to report all important data (residual DCIS and axillary node status). This will allow accurate meta-analysis with reference to the different definitions, even if the headline results are not completely harmonised in each trial report.

#### Type of breast cancer and prognostic and predictive factors

The biology of the type of breast cancer (e.g. ER-positive HER2-positive) will influence the pCR rate and often the effect size between a control arm and an experimental arm. The meta-analysis manuscript [5] shows clearly that the pCR rate increases the higher the biological risk (Fig. 3). In ER-positive low-grade cancers, achieving a pCR predicts an excellent outcome as it does in high-risk disease/biology, however, varying degrees of pathologic response has less prognostic value than in other subtypes. As an example, the HR for OS for those achieving pCR is 0.16 (95 % confidence interval [CI] 0.11–0.25) in triple-negative cancers but only 0.47 (95 % CI 0.21–1.07) for low grade ER-positive HER2-negative ones. This difference may relate to additional adjuvant hormonal treatments given to ER-positive patients as well as the different biology, for which a continued, although small, risk of relapse is present.

#### Small sample numbers

Neoadjuvant trials are powered for the primary endpoint of pCR and not for secondary endpoints such as relapse-free survival (RFS) and OS. On a purely statistical basis, unless the benefit in pCR rate is very large, there is unlikely to be a statistically significant result for the trial question in RFS and OS endpoints.

#### Adjuvant treatment

Any adjuvant treatment given after surgery in neoadjuvant trials is likely to influence longer-term outcomes, and clearly this effect will be greatest in patients who have not achieved a pCR at the time of surgery. Adjuvant treatments include 5–10 years of hormonal treatment in ER-positive disease, and anti-HER2 therapy in HER2-positive disease. Trials of new targeted agents in the adjuvant setting will also confound the relationship between pCR and longer-term outcomes, particularly if used to treat exclusively the non-pCR group. However, the outcome to be achieved here is for maximum patient benefit and our view is that patients who do not achieve a significant pathological response should have the opportunity to enter new clinical trials designed to test additional adjuvant treatments in non-responders. In the future it may be better to include mainly high-risk patients in neoadjuvant chemotherapy trials, identified by molecular markers of high-risk biology and predictive biomarkers of chemotherapy sensitivity. Patients with low-risk, ER-positive, HER2-negative cancers will all receive effective adjuvant hormonal treatments that influences the correlation between pathological response (especially for non-responders) and longer-term outcomes. For patients with low-risk but large ER-positive breast cancers, an alternative neoadjuvant management is to use hormonal treatments with a view to improving the rates of breast conservation.

#### Length of follow-up and interaction with type of breast cancer and prognosis

Length of follow-up in neo/adjuvant early breast cancer trials is a further factor influencing longer-term results. This has been reviewed for adjuvant breast cancer trial reporting [30], and the same arguments would hold for correlation between pCR and longer-term outcomes. In trials including both patients with ER-positive and ER-negative tumours, because the events for patients with ER-negative cancers tend to be mostly in the first 3 years, once the Kaplan–Meier curves have stabilised they will change little. Conversely, patients with ER-positive cancers continue to have a steady (albeit low) rate of events, which means that the longer the follow-up, the more widely the curves will separate. Therefore, length of follow-up at the time of analysis can influence the observed relationship between pCR and RFS and OS; more importantly, for a given improvement in pCR rate between trial arms, the magnitude and direction of this bias will depend on the specific trial population stratification.

### Definition of longer-term outcomes

We have talked generally about ‘longer-term outcome’ but the definition of this provides additional complexity when correlating with pCR. Disease-free survival commonly includes local recurrence in breast or local lymph nodes, the development of a new primary in the same or contralateral breast, the appearance of a second malignancy, and distant metastatic disease. RFS commonly includes all of the above except the development of a new second malignancy. For neoadjuvant trials and for correlation of pCR we are most interested in distant metastases free survival (DMFS), i.e. recurrence pertinent to the breast cancer we have treated. In terms of mortality indicators, breast cancer-specific survival (BCSS), i.e. death caused by breast cancer metastases, is more relevant than OS. In neoadjuvant trials, BCSS and OS are likely to be very similar because these trials generally do not include older patients with comorbidities that in larger adjuvant hormone trials will be causing additional deaths. An interesting demonstration of how much difference BCSS compared with OS makes to analysis of large patient numbers is the follow-on analysis of nearly 2,000 patients included in the intrinsic cluster breast cancer subtypes [31]. The longer the follow-up, the more effect from ‘all-cause mortality’ will be seen, which will impact to a greater degree in lower-risk patients. Therefore, with longer follow-up, non-cancer mortality disproportionately distorts the outcomes of lower-risk patients who do not die because of cancer and are relatively more exposed to these competing risks.

### The possible effect of clonal heterogeneity

There is increasing appreciation of clonal heterogeneity within ‘solid’ cancers that is probably present at the time of diagnosis in the primary lesion. In their seminal paper in 1979, Goldie and Coldman described mathematical models and hypotheses for spontaneous mutation rates in cancers, dependent on size, that were related to chemotherapy sensitivity and resistance [32]. It is possible, even likely, that clones which form the basis of micro-metastatic disease will be different from the dominant clones in the primary tumour. This is currently a field of intense activity in translational and scientific research. If metastatic disease were to be significantly different in terms of treatment sensitivity from the start, then any hoped-for correlation between the response of the primary tumour (pCR) and longer-term outcomes would be lost. This area of translational research will be greatly helped by analysis of circulating tumour cells (and circulating tumour DNA within neoadjuvant trials. Based on the work of Dawson et al. [33], we now include these translational analyses in all our neoadjuvant studies. We are optimistic that this will allow us to identify the small group of patients who have persistent micro-metastatic disease despite achieving a pCR, and the perhaps larger group of patients in whom, although pathological response is not complete, eradication of micro-metastatic disease is observed at the time of surgery. By means of this technology, over the next decade our ability to know at a molecular level what is happening in each individual patient will be enormously enhanced. Whilst all our dreams of personalising targeted therapy for our patients may not materialise, it may prove that circulating DNA and circulating tumour cell assays after neoadjuvant therapy and surgery may be helpful in defining those patients who require additional adjuvant treatment and those who do not, regardless of the response status of the primary tumour and lymph nodes after neoadjuvant treatment.

### Is bevacizumab a special case?: exploring the effect on the relationship between pCR and long-term cancer-related outcomes for a ‘pure’ angiogenesis inhibition

Reports of neo/adjuvant use of bevacizumab in early breast cancer have produced added complexity when examining the correlation between pCR and longer-term cancer-related outcomes. Four neoadjuvant trials, GeparQuinto [34], NSABP-B40 [35], CALGB 40603 (Alliance – Triple negative) [36] and ARTemis [4], all show a significant improvement in pCR with bevacizumab. For the purposes of comparative analysis, these trials exclude patients with HER2-positive tumours, which means that the biological differences (discussed in an earlier section) between the cohorts (i.e. ER-positive/HER2-negative and ER-negative/HER2-negative groups) are considerable. Whilst GeparQuinto, CALGB 40603 and ARTemis all show effect in the triple-negative group, the NSABP-B40 trial shows a significant effect in the ER-positive cohort. In terms of correlation with longer-term outcomes, the GeparQuinto group has now published on RFS and OS [8] and shows no correlation between the improved pCR rates and longer-term outcomes. In addition, two large adjuvant bevacizumab trials, BEATRICE (TNBC) and ECOG Study E5103 (HER2-negative), show no benefit from the addition of 12 months’ bevacizumab therapy to adjuvant chemotherapy [37, 38]. In our opinion, bevacizumab, acting as a ‘pure’ angiogenesis inhibitor, has the potential to improve rates of pCR in the primary tumour, which is angiogenesis-dependent, but has no such effect in the putatively angiogenesis-independent micro-metastatic disease, particularly in the bone marrow. Whilst adjuvant bevacizumab may delay the development of established metastatic disease during the 12 months of treatment, its lack of a direct anti-cancer effect makes it unlikely to eliminate micro-metastatic disease if it is present. The lack of efficacy in terms of RFS and OS in the adjuvant trials would support this hypothesis [37, 38]. At present, the report of longer-term outcomes from the NSABP-B40 [9] does not support this hypothesis because it demonstrates improved longer-term outcomes with bevacizumab in the ER-positive group when both neoadjuvant and adjuvant treatment is given. However, as discussed in the previous section, it may be too early to analyse long-term outcomes, particularly in the ER-positive/HER2-negative group.

The generic point we make here is the need to consider the mechanism of action of the anti-cancer agent being tested in the experimental arm as an important additional factor, with potential influence on the relationship between pCR and longer-term outcomes in neoadjuvant trials.

### Do the challenges to the correlation of pCR to longer-term outcomes invalidate neoadjuvant trials?

Clearly our answer to this is most definitely not. We base this opinion on a thorough review of all the literature, including the most recent publications of the exploration of the statistics behind the ALLTO/NeoALLTO controversy [39–41] and the detailed analyses of Hatzis and colleagues [42], which demonstrate the implications for future neoadjuvant trial designs using complex multifactorial modelling. There is a consensus, supported by evidence from Cortazar et al. [5] and Berruti et al. [6], and agreed by the FDA for licensing purposes, that neoadjuvant breast cancer trials will have most applicability in subtypes of breast cancer that have aggressive tumour biology and respond well to standard or targeted treatments. At present, the neoadjuvant literature has clearly identified triple-negative breast cancer and HER2-positive disease as fitting these criteria. In our view, ER-positive disease, which is low risk on account of tumour biology, is unlikely ever to achieve statistical correlation between pCR and longer-term outcomes because they are more likely to be affected by the multiple confounding factors described earlier.

**Fig. 4:**
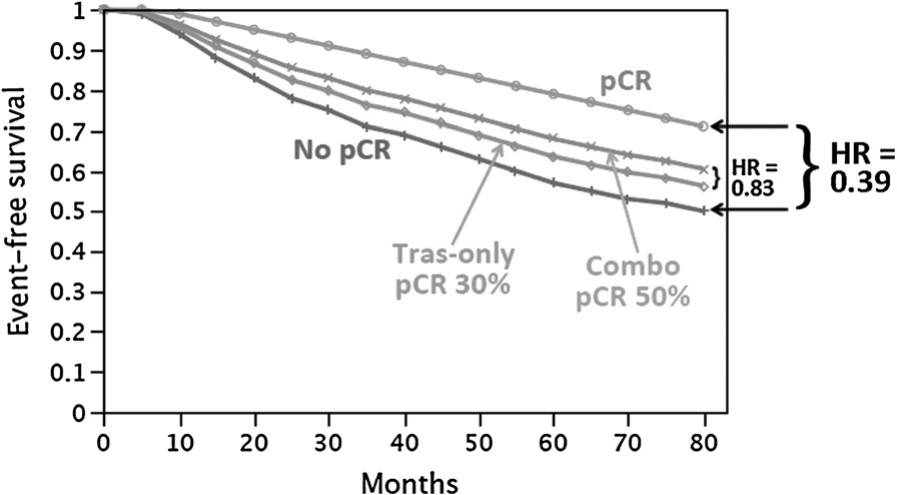
Expected event-free survival curves for trastuzumab as well as for the combination of trastuzumab and lapatinib are shown based on the NeoALTTO results. (Reproduced with permission from *Clinical Cancer Research*, DeMichele et al. [41]: License Number 3666990663029)

At present, the most notable outlier (even in high-risk groups) in terms of good correlation between pCR and longer-term outcomes is the NOAH trial [6, 10], which randomised patients to standard neoadjuvant chemotherapy with or without trastuzumab, which was continued after surgery in the experimental arm only. This trial was completed just before the paradigm-shifting adjuvant trastuzumab studies demonstrated the large benefit from adjuvant trastuzumab [43, 44]. We must remember that in ALLTO and NeoALLTO, the question asked by the trial was whether dual anti-HER2 therapy could improve on the impressive results of these initial trastuzumab studies. These ‘second order’ trials are always going to be more of a challenge because one would not necessarily expect more than a ‘marginal’ increase in benefit, unless patients have been selected at recruitment as poor responders to established therapy. In the event, Neo-ALLTO showed an absolute improvement of 20 % in pCR rates, and ALLTO narrowly missed statistical significance with an HR of 0.84 (97.5 %, CI 0.70–1.02), which was predicted to be HR 0.83 (Fig. 4), potentially owing to too few patients in the relevant comparisons. DeMichele and colleagues [41] detail compelling and robust arguments supporting the continued use of neoadjuvant trials in high-risk HER2-positive breast cancer. Theirs is an important generic review of the evidence given that the vast majority of future neoadjuvant trials in high-risk breast cancer will be asking questions about the benefit of new targeted agents in combination with standard effective therapies. In our view, the evidence cited above strongly supports the continuation of neoadjuvant trials as a way of identifying the potential benefit of new agents as quickly as possible. Perhaps more compelling is the opposite argument, that demonstrating lack of efficacy in a neoadjuvant trial should prevent the vast cost of testing the new agent in large adjuvant trials.

Hatzis and colleagues [42] have proposed that it is the baseline prognosis of the accrued patient population that has a major impact on the relationship between trial arm level survival and improvement in pCR. This makes some intuitive sense and implies that even large increases in pCR rate will translate into very modest improvement in survival if the baseline prognosis is already good. Both the BEATRICE and ALLTO adjuvant trials (of bevacizumab and dual anti-HER2 therapy respectively) demonstrate Hatzis’ modelling, because the trial arms had remarkable and unexpectedly good long-term survival outcome. In other words, to detect a long-term outcome effect between treatment arms with credible power, an unrealistically high pCR rate in the experimental arm would be required, as well as recruitment of more than 250 patients per treatment arm. In addition, Hatzis and colleagues used the NOAH trial to model the effect of using a primary endpoint that combines pCR with RCB down-staging as a more sensitive primary endpoint. They found that it correlated significantly with DMFS in their post hoc analysis of the NOAH trial.

### What about novel agents addressing unmet need in non-responders to neoadjuvant therapy?

Rather than focusing on the responders in neoadjuvant trials, what happens if we look at the non-responders in terms of pathological evaluation and try to improve their long-term outcomes? In neoadjuvant trials of high-risk breast cancer, although pCR rates are relatively high, there will still be a modest proportion who show no evidence of response. If we assign these patients to more neoadjuvant therapy, or additional adjuvant treatment to try and improve their outcomes and we succeed, then we remove any possibility of the correlation between pCR and longer-term outcome being significant. Would it go against good trial design and statistical planning to do so? Our view is that the most important outcome from a neoadjuvant trial in high-risk early breast cancer is the primary endpoint of pathological response and that to attempt to improve outcomes for non-responders would be a more valid endeavour, than to insist on within-trial validation with correlation of pathological response and longer-term outcomes.

### Summary

Neoadjuvant breast cancer trials have a great future but, in our opinion, with some modifications to their designs. Current neoadjuvant chemotherapy trials are not statistically powered (in terms of numbers) for longer-term outcomes. In addition, there is an emerging consensus that neoadjuvant chemotherapy trials are best focused on the high-risk population. Better definition of subgroups will mean more clarity in terms of the primary endpoint and perhaps more likelihood of finding a positive correlation with longer-term breast cancer-related outcomes. As clinical research into circulating DNA is carried out, the hope is that the definition of residual micro-metastatic disease or the lack of it will become more robust and therefore helpful in both the neoadjuvant and adjuvant settings.

Novel designs for neoadjuvant trials are emerging and the i-SPY group has led the development and implementation of adaptive Bayesian designs in neoadjuvant breast cancer [45]. This group has now moved two novel agents from phase 2 trials into i-SPY 3 trials [46], that will test in the standard setting the final stage of these elegant solutions to speeding up the introduction of new therapies for patients with early breast cancer. Our own group is developing Bayesian adaptive randomised designs to define ‘pairs’ of novel biomarkers and new agents [47]. Other adaptive designs in development will focus on high-risk patients who have not achieved a pathological response and these patients will be offered secondary randomisations into trials of novel therapy in the neoadjuvant and adjuvant setting. As these trials are developed, the ‘statistical’ consequences are inevitable; there will no longer be any possibility of positively correlating pathological response with longer-term outcomes, as we strive for better outcomes for patients not achieving a pathological response to first-line neoadjuvant treatment.
